# Forced Expiratory Volume in One Second Quotient (FEV1Q) as a Prognostic Factor in Amyotrophic Lateral Sclerosis Patients: Comparing Its Predictive Value to Other Lung Function Measurements

**DOI:** 10.7759/cureus.54176

**Published:** 2024-02-14

**Authors:** Carolina da Silva Alves, Tiago Barroso, António Gerardo, Tânia Almeida, Silvia Maduro, José Pedro Boléo-Tomé, Hedi Liberato

**Affiliations:** 1 Pulmonology Department, Hospital Professor Doutor Fernando Fonseca, Amadora, PRT; 2 Medical Oncology Department, Hospital de Santa Maria, Centro Hospitalar Universitário Lisboa Norte, Lisboa, PRT

**Keywords:** pcf, snip, mip, fvc, fev1, fev1q, amyotrophic lateral sclerosis

## Abstract

Introduction: Amyotrophic lateral sclerosis (ALS) is a neurodegenerative disorder affecting the first and second motor neurons. Forced vital capacity (FVC) and forced expiratory volume in one second (FEV1) have conventionally served as indicators of respiratory muscle strength. Recently, FEV1Q (FEV1 divided by the sex-specific first percentile values of absolute FEV1 in adults with lung disease) has been suggested as a predictor of mortality. While FVC has been utilized as a prognostic factor, FEV1Q has not yet been examined.

Methods: This retrospective unicenter study evaluated FEV1Q as a predictor of mortality in ALS patients, comparing its predictive efficacy with other measurements, including FEV1, FVC, sniff nasal inspiratory pressure, and maximal inspiratory pressure. The study utilized univariate analysis for each variable employing the Cox proportional hazards model to determine the statistical significance and predictive power of each measurement.

Results: Forty-five patients were included, female predominant (60%) and an average age at diagnosis of 69.2 ± 11 years. Almost all (95%) met the criteria for non-invasive ventilation (NIV) and initiated (93%) during the study period, a mean of 137 days after diagnosis. The mortality rate observed was 57%, occurring at a median of 398 days post-diagnosis. On average, patients underwent 1.7 pulmonary function tests, revealing a decline in various parameters, including FEV1, FEV1Q, and FVC. However, only FEV1Q was a statistically significant predictor of mortality (p < 0.0083) in a Cox regression analysis. A negative coefficient for FEV1Q indicated that higher values were associated with a reduced mortality risk, with an average FEV1Q of 2.68 observed at the time of death.

Conclusion: FEV1Q emerged as the only statistically significant predictor of mortality among the evaluated respiratory measurements in ALS patients. This study is the first to focus on applying FEV1Q in the clinical evaluation of ALS, marking an initial step in understanding its potential role in patient follow-up. However, further studies are needed before these findings can be incorporated into clinical practice.

## Introduction

Amyotrophic lateral sclerosis (ALS), a neurodegenerative disease, impacts both upper and lower motor neurons, leading to the weakening of limb, bulbar, thoracic, and abdominal muscles [1-3]. When ALS affects phrenic motor neurons, respiratory failure can occur due to diaphragm weakening and reduced lung functionality [3]. Pulmonary function decline is an important prognostic indicator in ALS and is used to determine when to start non-invasive ventilation (NIV) [1-4]. Forced vital capacity (FVC) has been used as an indirect marker of respiratory muscle strength, reported as a prognostic factor, and a recommended test for clinical trials [2,3,5]. However, spirometry's effectiveness in the early detection of inspiratory muscle weakness is limited [1,3]. A specific assessment of respiratory muscle strength could be accessed by the sniff nasal inspiratory pressure (SNIP) and maximum inspiratory pressure (MIP), which are more sensitive than FVC for diaphragm assessment [1-4].

ALS with cough reflex and bulbar function involvement contribute to respiratory morbidity and mortality due to an increased risk of respiratory infection. Measuring peak cough flow (PCF) is important in the management of the disease with cough assistance [1-3]. The main cause of death is respiratory insufficiency associated with pneumonia and diaphragm failure [3,6], usually 3 to 5 years after the onset of ALS [3].

The European Respiratory Society (ERS) and American Thoracic Society (ATS) recommend using standardized residuals (SRs), represented as z-scores, to assess whether an index falls outside the normal range in lung function tests. FEV1 has been consistently linked to all-cause mortality in the general population, although the underlying reasons for this association remain unclear. Notably, this relationship is even stronger when it comes to mortality from respiratory diseases associated with airflow obstruction [7].

In 2009, Miller and Pedersen introduced the concept of FEV1Q, which is calculated as FEV1 (forced expiratory volume in one second) divided by the sex-specific first percentile values of absolute FEV1 in adults with lung disease (0.4 liters for women and 0.5 liters for men) [7]. This approach provides the number of turnovers of FEV1 remaining above the minimal threshold for survival, rather than measuring the deviation from predicted values [7,8]. The same study found that FEV1Q outperforms the traditional FEV1% predicted value in predicting long-term survival [7-9]. FEV1Q values closer to 1 are associated with a greater risk of death [8]. Under normal conditions, FEV1Q decreases by approximately one unit every 18 years, but this rate accelerates to around one unit every 10 years for smokers and the elderly [8]. Changes in FEV1Q levels may signal a sudden decline in lung function, making it a valuable alternative for assessing significant changes in adults over time [8-10]. Notably, it does not specify the threshold that defines the minimal clinically significant difference in FEV1Q measurements [9].

In 2022, the interpretive strategies for routine lung function tests recommended by ERS and ATS shifted their focus to the evaluation of FEV1Q in adults concerning changes in lung function over time [8].

Currently, no studies have evaluated the FEV1Q as a predictor of mortality in neuromuscular diseases [9-11].

The primary objective of this study is to assess whether the FEV1Q is a statistically significant predictor of mortality in individuals diagnosed with ALS. Additionally, we have examined and compared the predictive potential of other pulmonary function parameters: FEV1, FVC, SNIP, and MIP, in ALS. Our secondary objective involves estimating the average rate of decline in FEV1Q over time by pooling data from all measurements obtained from the entire patient cohort.

It is noteworthy that this study represents a pioneering effort in assessing the utility of FEV1Q in patients with neuromuscular diseases. Preliminary data from the analysis of FEV1, FEV1Q, and FVC as predictors of mortality in ALS patients were presented as a meeting abstract at the 2023 SPP - Congress of the Portuguese Society of Pulmonology on November 10, 2023, in Portugal.

## Materials and methods

Study population and design

We performed a retrospective, single-center study conducted at Hospital Professor Doutor Fernando Fonseca, Portugal. ALS patients were followed by a multidisciplinary team that included Pulmonology, Neurology, Gastroenterology, Nutrition, Physical Medicine, and Palliative Care. They underwent lung function tests including spirometry, plethysmography, SNIP, MIP, and PCF. Patients underwent these tests at the time of diagnosis and on the same day during their follow-up visits (monthly or every three to six months, depending on the disease's progression) until collaboration became unfeasible due to muscle weakness.

We analyzed clinical and demographic variables, including age, sex assigned at birth, date of diagnosis, date of death, number of lung function tests per patient, the use of NIV, the use of mechanically assisted cough, and the use of percutaneous endoscopic gastrostomy. When applicable, we also described the time intervals between diagnosis and death, initiation of NIV post-diagnosis, and duration until death.

This study adheres to the Declaration of Helsinki and obtained approval from the institutional ethics committee of Hospital Professor Doutor Fernando Fonseca (registration number 102/2023). Written informed consent was waived.

Pulmonary function tests

Baseline values of FEV1, FVC, SNIP, MIP, and PCF were obtained from regularly performed lung function tests. Trained operators conducted pulmonary function maneuvers according to recent ERS/ATS recommendations for performance and interpretive strategies for lung function tests [7], using MasterScreen Body™ (CareFusion, Germany) for spirometry and plethysmography, and Datospir Peak-10™ (Sibelmed, Spain) for PCF. All tests were conducted in a seated position, with the use of a nose clip when applicable. FEV1Q was calculated as FEV1 divided by the sex-specific first percentile of the FEV1 distribution (0.4 L for females and 0.5 L for males).

The decline over time in the described lung function test variables was analyzed.

Statistical analysis and data visualization

To assess each of the pulmonary function tests as a predictor of mortality, each result from the lung function tests was considered an independent value, treating measurements from the same patient and different patients alike. Each measurement was viewed as a continuous variable. To assess the impact of each respiratory function test on patient survival, we conducted a univariate analysis for each variable using the Cox proportional hazards model with right-censored data, with the variable as the sole mortality predictor. We chose to use an alpha level of 0.05 as the threshold for statistical significance, which was adjusted for multiple comparisons using the Bonferroni correction. As we conducted six significance tests (one for each respiratory function test), this significance threshold corresponds to a p-value < 0.0083 (0.05 divided by 6). The remaining clinical and demographic variables were analyzed descriptively.

To visualize the relationship between the decline in FEV1Q and patient survival, we created a plot of FEV1Q values as a function of the time until the patient's death. Values measured from patients who were still alive at the time of data collection were excluded. Subsequently, we performed a linear regression analysis on these values, treating them as independent observations, to assess the expected rate of FEV1Q decline and the anticipated FEV1Q value at the time of death.

We used a custom program written in the Python programming language [12]. The lifelines package [13] was used for the survival analysis (Cox regression using the proportional hazards model), and data visualization was done using the Matplotlib package [14].

## Results

During the study period, we identified 47 patients with ALS. Two patients could not participate in the pulmonary function tests due to having been diagnosed at an advanced stage of the disease, resulting in the inclusion of 45 patients in the study. Most of them were female (60%, n=27), with a mean age at diagnosis of 69.2 ± 11 years. Notably, 36% (n=16) of patients had bulbar involvement at diagnosis (Table 1).

**Table 1:** Each row represents a single univariate analysis for a specific variable as a predictor of mortality. Given that we carried out six individual comparisons (one for each predictor variable), we applied the Bonferroni correction to establish a threshold of alpha = 0.05 / 6 = 0.0833 for statistical significance. This corresponds to an alpha level of 0.0.0083 for a single comparison, equivalent to the use of 99.2% confidence intervals instead of the standard 95% confidence interval for a single comparison. All confidence intervals are two-tailed. The coefficient that is statistically different from zero (or, equivalently, the exponential of the coefficient that is statistically different from 1) is denoted with an asterisk (*). Labels include 'coef,' representing the coefficient in the Cox regression for the specific analysis; 'exp(coef)' representing the exponential of the coefficient directly related to the hazard ratio for the predictor's impact on patient survival; and 'CI,' indicating the confidence interval for the given parameter. FEV1Q - corrected forced expiratory volume in the 1st second, FEV1 - forced expiratory volume in the 1st second, FVC - forced vital capacity, MIP - maximum inspiratory pressure, SNIP - sniff nasal inspiratory pressure, PCF - peak cough flow.

Variable	coef	coef CI 99.2%	exp(coef)	exp(coef) CI 99.2%	p
FEV1Q	-0.426	[-0.791, -0.061]	0.653	[0.453, 0.940]	0.002*
FEV1	-0.473	[-0.974, 0.027]	0.623	[0.378, 1.027]	0.013
FVC	-0.312	[-0.730, 0.105]	0.732	[0.482, 1.111]	0.048
MIP	-0.007	[-0.025, 0.011]	0.993	[0.975, 1.011]	0.306
SNIP	-0.024	[-0.048, 0.000]	0.977	[0.953, 1.000]	0.010
PCF	-0.005	[-0.010, 0.000]	0.995	[0.990, 1.000]	0.009

**Figure 1:**
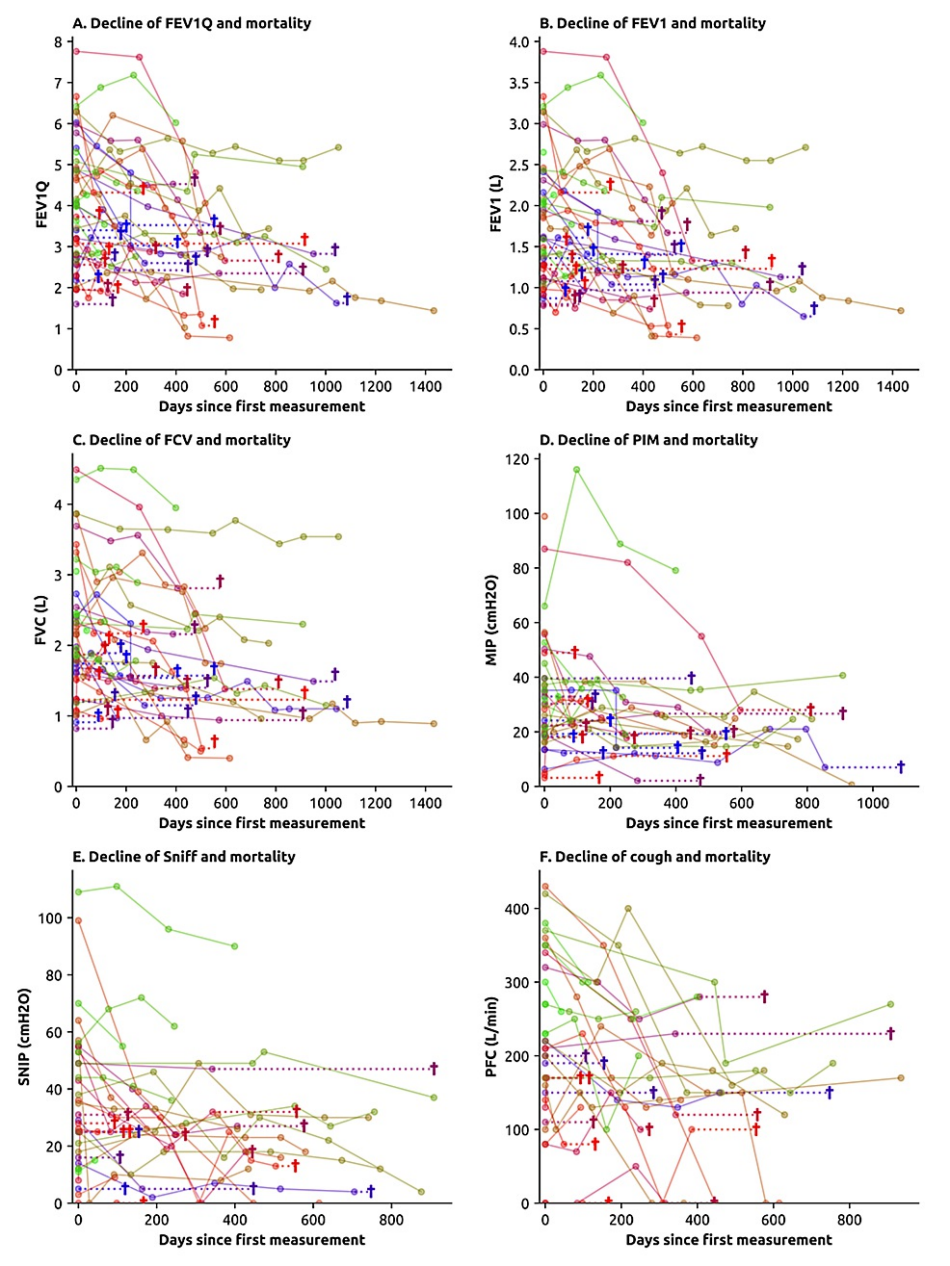
This figure illustrates the progression of various pulmonary function tests for each individual patient, along with the time of death when available. Each patient's data is represented using a unique color. Each data point represents a distinct measurement, with lines connecting data points from the same patient. When the date of death is known (i.e., when not censored), it is denoted by a cross (†), connected to the last pulmonary function test with a dashed line. These plots emphasize the overall trend of pulmonary volumes and airflow measurements declining over time due to deteriorating respiratory muscle function. The following pulmonary function tests are depicted: (A) FEV1Q, (B) FEV1, (C) FVC, (D) PIM, (E) SNIP, and (F) PCF. FEV1Q - corrected forced expiratory volume in the 1st second, FEV1 - forced expiratory volume in the 1st second, FVC - forced vital capacity, MIP - maximum inspiratory pressure, SNIP - sniff nasal inspiratory pressure, PCF - peak cough flow.

It was found that 95% (n=43) of the patients met the criteria for NIV usage, with 93% (n=40) of these starting NIV during the study period (a median of 137 days after diagnosis). In addition, 91% (n=41) started mechanical insufflator-exsufflator (MI-E), and 36% (n=16) required percutaneous endoscopic gastrostomy. The mortality rate in the study period was 57% (n=26), with patients dying at a median of 398 days (IQR 216.5-1218) after diagnosis and 99 days (IQR 58-366) after the last pulmonary function test. Of the patients who died, 56% (n=12) had bulbar involvement and 8% (n=2) refused to undergo NIV. The remaining patients underwent NIV a median of 192 days before the time of death. Regarding pulmonary function evaluation, patients underwent on average 1.7 tests during the follow-up period. A decline was observed in FEV1, FEV1Q, FVC, and other parameters, as shown in Figure 1, where the lung function measurements were plotted for each patient as a function of time, together with the date of death, when applicable.

**Figure 2:**
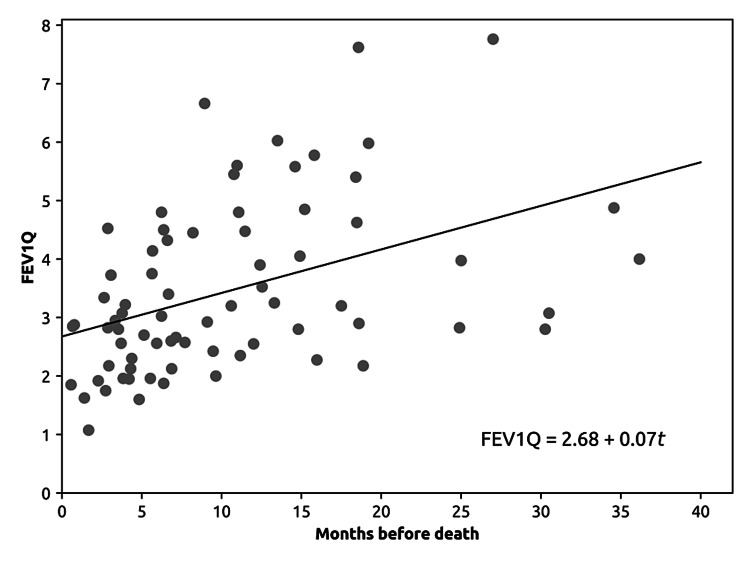
Linear regression of pooled FEV1Q values over time for all dead patients. Patients who were still alive at the time of data collection were excluded from this plot because they could not be used to evaluate the intercept of the linear regression. FEV1Q is plotted against the time until death, setting the value of FEV1Q at time 0 as the value at the time of death. With this parameterization, the intercept of the curve represents the average FEV1Q value at the time of death (2.68), and the slope of the curve (0.07) indicates the monthly rate of decrease. This model suggests that, on average, FEV1Q decreases by 0.07 per month, and death occurs when FEV1Q reaches 2.68. FEV1Q - corrected forced expiratory volume in the 1st second

At the chosen level of statistical significance, of all lung function measurements, only FEV1Q was a statistically significant predictor of patient mortality (Table 1), with a coefficient in the Cox regression of -0.426 (99.2% CI -0.791, -0.061). In the Cox regression, a negative coefficient means that high values of FEV1Q protect from the risk of death, while low values of FEV1Q increase the risk of death. The remaining lung function tests were not significant predictors of mortality in our sample with our chosen significance level, with the confidence intervals for the regression coefficients including zero (Table 1). FEV1Q emerged as a statistically significant predictor of mortality (p < 0.0083). The FEV1Q decreased by 0.7 per month, and death occurred, on average, when the FEV1Q was 2.68 (Figure 2).

## Discussion

This study investigated the association between lung function and mortality in patients with ALS and found that, among the evaluated variables, only FEV1Q was a statistically significant predictor at the 0.05 level of significance.

In multiple studies, it has been demonstrated that measuring basal FVC has prognostic significance in ALS [5]. Most of the evidence is derived from univariate and/or multivariate Cox models that found FVC as a survival prognostic factor [2,15-17]. However, studies have already indicated that FEV1 might be a more accurate predictor of mortality than FVC, particularly in patients with obstructive airflow conditions [11,18,19].

In ALS, neuromuscular weakness usually manifests as a complex restriction (CR) pattern [8,20]. As the neuromuscular weakness worsens, the chest muscles become unable to overcome the elasticity of the chest wall, preventing exhalation below the chest wall's equilibrium point. This leads to an increased residual volume (RV) and a more significant reduction in FVC compared to total lung capacity (TLC). As the severity of the CR pattern progresses, the RV progressively increases as well [8,20].

Additionally, the FVC may remain unaffected until profound muscle weakness sets in, owing to the sigmoid nature of lung pressure-volume curves (21). Consequently, FEV1 may better assess the risk of mortality not only for patients with obstructive conditions but also for those with restrictive diseases, such as ALS [1-4].

Miller and Pedersen discovered that FEV1Q was a more reliable predictor of mortality compared to other expressions of FEV1 [7]. This finding was supported by studies conducted among chronic obstructive pulmonary disease (COPD) patients by Bhatta et al. and among asthmatic patients by Pedone et al. [21,22]. FEV1Q was also identified as a more accurate predictor for severe acute exacerbations (including frequent exacerbations) and mortality risk in COPD patients compared to other FEV1 variations, such as predicted value, ratios of FEV1 over height squared (FEV1·Ht-2), and over height cubed (FEV1·Ht-3) [23].

These findings suggest that the severity of the obstructive disease seems to be more closely associated with the deviation of an individual's FEV1 from the bottom line rather than its deviation from a predicted value [21].

The refinement that FEV1Q introduces to the FEV1, by correcting such values for the minimum viable FEV1 values for a person of the same sex [7,8] probably contributes to making it a better predictor of mortality, including in ALS patients.

Furthermore, the FEV1Q value near death in our sample approached 2 and not 1 as described in the literature [8]. It was observed that from diagnosis to death, the FEV1Q decreased monthly, which may suggest that the loss of stability of this marker could correspond to a progressive worsening of ALS. However, studies using FEV1Q do not mention a threshold that defines the minimal clinically significant difference in FEV1Q measurements [9].

Considering the limited sensitivity of spirometry [3], a more comprehensive assessment of respiratory muscle strength is usually performed in clinical practice. SNIP is a sensitive indicator of respiratory muscle strength, can be recorded even in advanced disease when spirometric values may not be feasible, and provides valuable prognostic information [24]. Various SNIP thresholds have been proposed for prognostic purposes and to initiate NIV [3]. For example, a study found that a value below 40 cmH2O predicted death within 6 months [24]. Recent research points to significant changes in SNIP values within 3 months before starting NIV, with a cutoff of 34 cmH2O predicting death or tracheostomy within a year [25]. Another study suggested a higher threshold of 45 cmH2O [26]. Our study may provide additional information for the prediction of survival, using a different parameter.

PCF has also been identified as a prognostic factor for mortality in ALS patients [27]. According to Bach et al., PCF values below 270 L/min and 160 L/min are clinically significant, indicating an inability to cough effectively and clear airway secretions, respectively [28]. This might increase the risk of respiratory infections.

Several potential limitations should be acknowledged in this study. First, the small sample size poses a challenge as it may restrict the translation of the findings to clinical practice. The small sample size might mean that potentially significant relations between pulmonary function tests other than FEV1Q might have been missed. Similarly, the fact that this is a unicentric study compromises the external validity of the data and makes it harder to generalize to other populations. Also, the presence of bulbar muscle weakness in some patients introduces an additional limitation. These individuals might encounter difficulties in achieving a secure seal around the mouthpiece during measurements, which could potentially result in recorded values that do not precisely reflect their true respiratory muscle strength [24].

This study marks the initial phase in understanding the use of FEV1Q in the follow-up of ALS patients. These results should be replicated in a larger and more diverse population to validate FEV1Q as a predictor of mortality in ALS patients and to understand what the role of this measurement could be in clinical practice.

## Conclusions

The FEV1Q emerged as the most reliable predictor of mortality among the variables analyzed. In our sample, it was the sole statistically significant predictor of mortality at a significance level of 0.05 (adjusted for multiple comparisons). FEV1Q decreased monthly and approached 2 by the time of death. These analyses should be replicated in a larger and more diverse population before these results can be applied in clinical practice.
